# A Paper Considering Questions in Regard to Alcoholic Stimulants

**Published:** 1873-02-15

**Authors:** Chas. W. Earle


### Original Communications.

### A PAPER CONSIDERING QUESTIONS IN REGARD TO ALCOHOLIC STIMULANTS.

BY CHAS. W. EARLE, M.D.

*Read before the Chicago Medical Society.**

THE CAUSE OF ALCOHOLISM.

The condition known as alcoholism is produced by certain causes. These causes are *exciting* or *predisposing*. The exciting cause, which is by far the most frequent, and really the only one, is the circulation of blood highly charged with alcohol, affecting first and primarily the cerebro-spinal centers.

As the habit becomes confirmed, probably not an organ or tissue of the body escapes the deleterious effects of the article. The effect of alcohol on the nervous system, as we now understand it, and the manner in which it damages the other tissues through this, I hope to bring out in another part of this paper.

Of predisposing causes, I place at the head of the list anything which tempts a person to take alcoholic stimulants:—prostrating diseases, which reduce the system to an abnormal condition, in the course of which disease stimulants may have been prescribed; occupations of all kinds which expose the person to the extremes of heat and cold; which prevent sleep at the proper time; which surround the man with an atmosphere filled with noxious vapors, or impurities of any kind.

That occupation which renders night work necessary I regard as most dangerous to one who has ever used alcoholic stimulants. It is exceedingly difficult for one to reform who is engaged in this kind of work; and I would most earnestly advise that man who is trying to stop the use of alcoholic stimulants to change his occupation, so that whatever of work he has to do may be done during the day.

Of one thousand men admitted to the Washingtonian Home of Chicago, since 1865 to date, December, 1872, fourteen per cent. have been men whose work has been largely done during the night. Their occupations are as follows, enumerated according to the frequency of admissions: printers, traveling men, actors, editors, railroad men.

A large number of printers, at one time and another, are engaged on morning papers, working most of the night. Traveling men, or agents, very often sell goods during the day, and make the next business centre during the night. The actor always works till late; while the work of editors and railroad men, in many cases, is constant and exhaustive night labor. Combined with this, these men, in many instances, are deprived of home-influence, have an irregular and changeable diet, and are exposed to all the vicissitudes of weather.

On the other hand, however, of one thousand men admitted to the same institution, but two per cent. have been carpenters. These men work from eight to ten hours during the day, have regular meals, mingle with good society, if they will select it, and sleep soundly during the night.

THE PHYSIOLOGICAL PLACE OF ALCOHOL.

The effect of alcohol on the different tissues of the system has been the subject of much controversy for years. Used in large quantities it is unqualifiedly a poison, and not a food. Under ordinary circumstances, when the body is properly and sufficiently nourished, it does harm by even a moderate introduction into the system.

When, from some extraordinary circumstances, a person is exposed to severe labor or exposure, with an *insufficiency* of food, alcohol may, for a very short time, temporarily restore the powers of the body, but can never, in any way, take the place of assimilable nutriments.

In the treatment of disease, as a physician, I believe that occasionally the time does occur when stimulants are absolutely indis-

* This article was also read before the inmates of the Washingtonian Home, who, believing that it would do good to any wishing to reform, unanimously requested its publication. This will account for the exhortations at the close of the article. It is also believed to contain some things of interest to the profession.

THE AUTHOR.

pensable, and that in withholding them we commit an error just as reprehensible as it would be for a physician to prescribe a moderate amount of whisky for a period of six or eight days to every person getting over a moderate drinking-bout. In rare cases alcohol does good—for a brief time. Under no circumstances or conditions can good accrue when used for any length of time.

I close my observations on this part of my subject by quoting from the testimony of Dr. Hayes, the celebrated Arctic explorer. He says:

"While fresh animal food, and especially fat, is absolutely essential to the inhabitants and travelers in Arctic countries, alcohol is, in almost every shape, not only completely useless but positively injurious." *

THE EFFECT OF ALCOHOL ON THE TISSUES.

The continued use of alcohol produces certain local effects on different kinds of tissue. These lesions have been clearly proven by Dr. Ogston's *post-mortem* inspections, and may be enumerated as follows: †

1st. The nerve-centres present the greatest change.

2d. Change in the respiratory organs.

3d. The liver, and other glandular organs.

4th. The heart and large arteries.

5th. The kidneys.

6th. The alimentary canal.

These abnormal changes are seen best by the microscope, and consist of accumulations of fat, of degeneration of the arteries, of induration or hardening of nerve-matter, by a state of the system in which the general nutrition of the body suffers, inducing in every respect a bad state of health, until at last we have the drunkard's *cachexia*—the drunkard's own peculiar constitution.

The *immediate* effect of alcohol on nerve-tissue is perhaps most noticeable—it certainly is in acute cases.

If you should take one of the inferior animals, chloroform it, and dissect out one of its nerves and surround it with alcohol, it would be paralyzed.

Unable to transmit ordinary nerve-force, alcohol has poisoned it. The inordinate use of spirits affects the nervous tissues, as described above, in the following order:

1st. Paralysis of spinal nerves, as shown by the impairment of muscular sense in the extremities; numbness of the integuments, etc.

2d. Partial loss of sensibility in the integuments and mucous membrane of the face; numbness of lips; want of usual sensibility in the anterior part of the tongue. It is the tri-facial or large portion of the 5th nerve now affected. The vaso-motor branch of this nerve is relaxed, as evinced by the congested and palsied condition of the face.

3d. The cerebral hemispheres are affected, and intellection of all kind is impaired. The man has trouble to articulate words; he can still taste; the special nerves to the tongue, such as the *chorda tympani, and glosso pharyngeal, to the anterior part, and the lingual of the fifth to the posterior are intact; but the nerve of motion, the sublingual, or hypoglossal, has lost its power—has been paralyzed.

Finally, the medulla oblongata is reached; breathing is labored; the nerves absolutely necessary for the maintenance of life are affected, and at last the nerves supplying the heart are paralyzed, and the scene closes—death is the result.†

This, gentlemen, is the immediate effect of alcohol as a poison. Of course, not every one who uses the article moderately arrives at this dire result. I have depicted its immediate result as a poison. In many people the effect of a *small* amount of whisky is far from paralyzing. They talk more rapidly, and louder than ever before. They are capable of doing a large amount of physical labor, and endure, for a short time, almost any fatigue or exposure. The permanent effect on the tissues is being made, however, and sooner or later, in some disease of the respiratory organs—in some gland, in the

* Hayes' "Observations upon the Relations existing between Food and the Capabilities of Men to Resist Low Temperature." —*Am. Journal Med. Sciences*, July, 1859, p. 117.

† Aitken's "Practice of Medicine," Vol. I., p. 784.

* See Flint's "Physiology," Vol. IV., p. 156.

† See article on Alcoholism in Reynolds' "System of Medicine."

heart or kidneys, and rarely in the alimentary canal—it becomes manifest.

It is the popular notion that the stomach becomes diseased very early in a subject of alcoholism. Persons addicted to the use of stimulants are sometimes seized with a sort of mania, and imagine their stomachs are ulcerated and eaten. This I believe to be rarely the case. The mucus membrane of the stomach may, and does, become congested; the food coagulates before it is properly digested; the walls of the stomach may become thickened, but rarely ulcerated or eaten. The poison acts on other tissues more important, the disintegration of which cause the frightful train of diseases which I have already mentioned.

IS DELIRIUM TREMENS CAUSED BY THE SUSPENSION OR CONTINUANCE OF ALCOHOLIC STIMULANTS?

Inebriation, delirium tremens, mania a potu, and alcoholism—all very vague terms—convey to us the condition in which a man or woman is found during the use, or after the suspension, of alcoholic stimulants.

Delirium tremens is defined by a majority of the authors as an affection *following* the withdrawal of alcoholic drink or other stimulants. I have not been satisfied with this definition since I have more especially studied this particular disease. Unless I am very much mistaken, delirium tremens oft-times overtakes its victim in the midst of his excesses, while the system is saturated with the vile compound, and replenished with frequent and liberal potations. Upon looking up the literature of the subject I find that my idea in this respect is not at all original, as a Dr. Ware, in 1831, placed the same on record; and Reynolds, in his "System of Medicine," quotes from him, and evidently accepts the same.

Flint says it is a mooted question—and both explanations are correct—the disease sometimes making its appearance when the customary stimulant is suspended, and at other times, notwithstanding the continued use of the habitual amount of alcohol.

This is the first question to which I wish to call the attention of the Society. It is important to us in the treatment of our patients. It is important to the patient. The physician who believes delirium tremens the result of the withdrawal of the accustomed stimulant will be very apt to prescribe it, while the patient who shares in this belief will very likely either treat himself, or be treated by his friends, with a liberal amount of whisky for several days following the acute attack.

Any theory advanced by one not accustomed to the use of stimulants must fall to the ground when we have the testimony of those perfectly competent, who speak from experience.

The writer of this article has had the opportunity to receive from gentlemen of high culture, speaking from experience, ample testimony on this point.

It is unmistakably correct that the condition known as delirium tremens is not always due to the withdrawal of the stimulant. It makes its appearance at times when the system is fully charged with the poison—indeed while the stomach is being replenished—and at other times after the absolute withdrawal, be it voluntary or otherwise, on the part of the individual.

IS THE APPETITE FOR ALCOHOLIC STIMULANTS A DISEASE?

It is not the diseased condition alone with which we find a man accustomed to the habitual use of intoxicating drink the unfortunate subject, that we have to do; neither does it suffice for us to find its physiological place; there are other questions, important to every inhabitant of our land, which must be considered.

Why *do* men drink? Is this habit, which becomes the ruin of so many young men, blasts the hopes of families, and threatens the safety of our country, *hereditary?* or is it a disease?

Can a man drink all his lifetime and say that he cannot help it, because his parents used whisky?

Should a man be permitted to become intoxicated every few days, and be taught that he has no responsibility in the matter because he has a disease?

Is it right for the medical profession, for the ministry, for all the charitably-disposed people, to maintain that after a person has indulged himself with whisky or tobacco for years, until he feels uncomfortable when not under its effect, that the man has a disease which should be treated by some medicinal agent?

To all of these questions, at present, I say no.

During the past few years it has been quite popular to speak of those addicted to the use of alcoholic stimulants as suffering from a disease. Eminent men in the medical profession have expressed their belief in this direction; and not a few articles have been written on Intemperance as a Disease. No one can believe more thoroughly than I do that the long-continued use of alcohol produces many lesions in various organs in the body; but that the appetite for whisky is a disease, *sui generis*, does not seem to me rational.

A disease generally produces such an effect that its victim cannot throw it off in an instant. A person with typhoid fever cannot throw it from his system in a day; nor can the victim of consumption, worn down by it for years, by a simple effort of the will change his entire constitution and become a sound man. But if a man who for twenty-five years has not been free from the influence of liquor, in an hour makes up his mind that he will never again touch the article—if he will take the trouble to sever himself from his old associates, and not run headlong into temptation—he will soon be free from the so-called disease. This is certainly totally unlike any other disease with which I am familiar.

Again, persons afflicted with a disease generally are conscious of it. They believe and are conscious that they have some physical difficulty. But a man who truly and honestly wishes to reform from the use of alcoholic stimulants, and is willing to abandon his whisky-associates, and earnestly desires to become better in every way, will not tell you that he believes himself the victim of a disease. A man, however, not fully decided as to whether he wants to reform or not, with friends anxious to conceal his habit, and to furnish an excuse for him when he does break out, will very eagerly conclude with any one that it is a terrible disease.

The effect of alcohol on the tissues is *most damaging*. The appetite becomes terrific. But, gentlemen, I have yet to see the man who, having candidly and prayerfully made up his mind to stop the habit, who is willing to keep away from saloons and from those who would entice him to drink, that cannot reform.

If it was a disease, a man who had indulged in the habit for years and years, but who was willing to conform to the resolutions indicated, would have infinitely harder work to reform than one who had drank but a month. And yet we see men who have been addicted to the habit for years reform—stop at once—while the man who has been a drunkard but for a month yields to the seductive glass time after time.

This does not act to me like a disease. The difference is just here. One man makes up his mind fairly and squarely that he will stop—avoids his former companions, shuns saloons, and in every respect tries to become a better and wiser man;—the other does not make up his mind that he really and truly wants to reform—is not yet convinced that it is important, but will do so if convenient, or necessary to hold a situation; still mingles with associates who drink; visits saloons for cigars or mineral-water, and, in no respect whatever, tries to become a better man—is still profane, profligate and unruly. The one reforms; the other does not; and the one reformed has a thousand times more the disease (if there was such a thing) than the one who still persists in his course.

Such being the facts, it is very difficult for me to class either the habit of drinking or appetite for alcoholic drink as disease.

### IS THE APPETITE FOR ALCOHOLIC DRINK HEREDITARY?

I go still further: I do not believe that the habit of drinking, or the appetite for alcohol, is transmitted by our ancestors. And yet a great many people do believe this; and the remark has been made to me that it was "No use trying to stop drinking, for it was in the family—the father and grandfather, and as far back as the history could be traced, all drank — and it is natural for me. I can't stop!"

Now, it is undoubtedly true that a number of children of those addicted to the use of alcohol, are afflicted with insanity, become idiots, or have a tendency to epilepsy. It is also true that many children who were unfortunate in having intemperate parents, have grown up and been honest and industrious, never tasting a drop of liquor. Again, too, how many there are, raised in a family surrounded by everything calculated to make one good and true—watched by a father and mother anxious to do everything to develop a noble man—who also fall. As a general thing parents give to their offspring certain tendencies of their own constitutions, and in some cases a diseased system. These are all indubitable facts. But it appears to me, however, in the cases we are now considering, it is not a desire or appetite, or even habit, which is inherited, but rather a peculiar nervous system, making both bodily and mental labor or trouble hard to be borne, and inducing in the subject, if his early education be neglected, a feeling for some kind of stimulus. A proper training while young, with a rigid hygienic condition, will eradicate the difficulty. Even if this is neglected, the man need not despair—a resolute will, good associates, and avoidance of everything tempting, will accomplish it.

What I have wanted to say, and place on record, in the concluding remarks of my article, is this: There is no physical cause why a man should drink intoxicating liquors; and secondly, if a man has formed the habit, if he wants to reform he can do it.

There is something better for every man than to die a drunkard. And although the man may seem lost, the hopes and expectations of friends long since blasted, the victim and his friends completely discouraged—all this may obtain, but the man may reform. I believe it to be absolutely wrong to argue otherwise; and the doctrine that a man has some physical difficulty in the way of reformation, seems to me pernicious and dangerous in the extreme.

It has seemed to me that about five or six things were necessary for reformation:

1st. A man must thoroughly make up his mind that he wants to reform; he must mean business—*nulla vestigia retrorsum* (no steps backward.)

2d. He must come fairly to the conclusion that no back-set in business, no disappointment of any kind, is going to be forever rectified by returning to his former habits.

3d. He must make up his mind that, in addition to reforming from one habit, he will try to become better in every way possible.

4th. That he will avoid saloons as he would a bottomless pit.

5th. He must make up his mind to choose moral and temperate companions, avoiding those of opposite habits.

A man firmly pledged to these resolutions, thoroughly in earnest, will succeed. He must always be on his guard—not over-sanguine in his own strength, but recognizing a Divine Helper, always near to the unfortunate wishing to do better.

Thus resolving and performing, every man may go forward, gaining strength day by day; becoming more and more a man; regaining that which was lost; becoming established in the confidence of his fellow-men; bringing light and joy again to his own home; his body purified; his hopes and aspirations elevated; and the end is crowned with triumph in a perfected manhood.

